# A look at the performance of barrel and wedge assembly in cable bolts applications

**DOI:** 10.1038/s41598-024-54999-6

**Published:** 2024-02-23

**Authors:** Ashkan Rastegarmanesh, Ali Mirzaghorbanali, Kevin McDougall, Naj Aziz, Sina Anzanpour, Hadi Nourizadeh, Abbas Taheri

**Affiliations:** 1 Centre for Future Materials, University of Southern Queensland, Toowoomba, Australia; 2 School of Civil, Mining and Environmental Engineering, University of Wollongong, Wollongong, Australia; 3 The Robert M. Buchan Department of Mining, Queen’s University, Kingston, Canada

**Keywords:** Cable bolt, Barrel and wedge, Termination method, Pull out, Civil engineering, Structural geology

## Abstract

Pretensioning is one of the most common practices in cable bolting. A barrel and wedge is typically used in the free end of the cable to hold the pretension load. This study investigates the performance of barrel and wedge in cable bolt large-scale laboratory pull out tests. Twenty-five experiments have been completed containing various barrel and wedge and cable sizes under different loading conditions, namely monotonic and cyclic. The results indicated barrel and wedges undergo constant displacement throughout the experiment. The cyclic tests suggest that the barrel and wedge assembly displacement are almost entirely non-reversible. Two distinct behaviours, namely exponential and deflection point based, were observed. The study concludes that barrel and wedge assemblies can significantly influence the performance of cable bolts under axial load.

## Introduction

**Figure 1:**
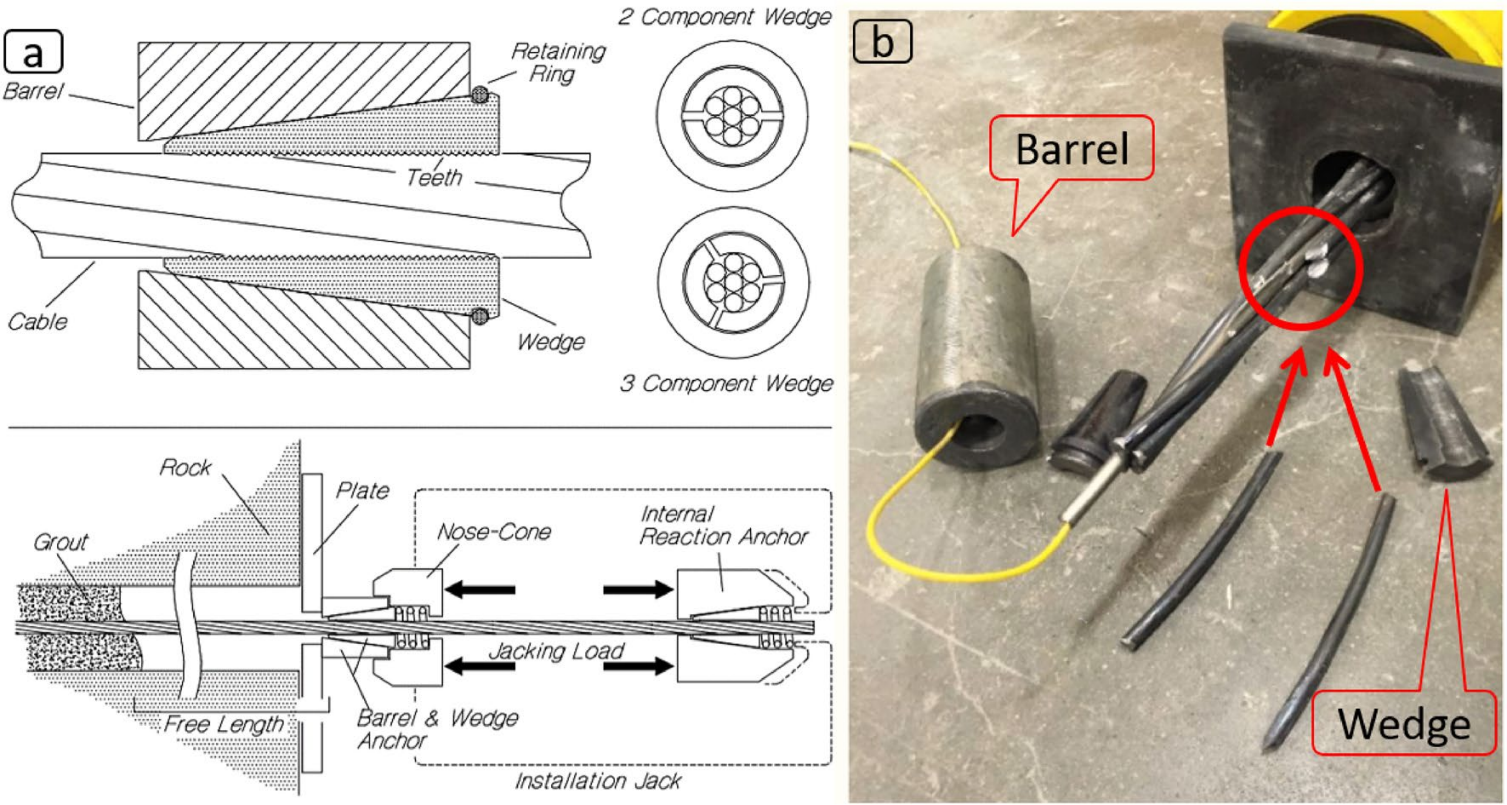
(**a**) Barrel and wedge design and activation method in the field^2^ after Thompson^3^, (**b**) failure at barrel and wedge^4^.

Barrel and wedge is the most popular termination method for cable bolts, thus an inseparable part of cable bolting. These elements have been used for almost a century and garnered a reputation for being robust and reliable. The overall concept of the barrel and wedge assembly (as seen in Fig. 1) is that the wedge comes in contact with the cable while resting inside the barrel. The outside surface of the wedge and the inside surface of the barrel are tapered. As the load (vertical displacement) is transferred to the barrel, confining pressure increases on the wedge, resulting in squeezing the cable harder. To minimize the slippage of the wedge relative to the cable, the inside surface of the wedge is indented (has teeth), so the wedge can “bite” into the cable material.

According to Jennmar^1^, the typical axial load capacity for a cable is around 10–20% lower at the barrel and wedge location. This is due to the enormous squeezing load that is caused between the wedge and the cable, which crushes the strands, as reported by Forbes and Vlachopoulos 2016 (Fig. 1b).

Lardner and Littlejohn^5^, as part of the ISRM suggested method of rock anchorage testing, provided a guideline for in situ testing of tendons. It was noted that the measurement of the ram stroke in pull out test with a grip (barrel and wedge) is overestimated due to the slippage of the grip. As a result, this factor should be compensated, or a point measurement method (such as the cable end) should be adopted.

Chen^6^ compared the performance of different termination methods in the pull out test. Three techniques, namely barrel and wedge, end plate, and an internally threaded plate screwed to the top of the externally threaded anchor tube, were compared for MW9 cable. The result showed that no significant difference exists among these techniques, and as long as the anchor tube is designed and utilized correctly, the results are almost similar in the end.

**Figure 2:**
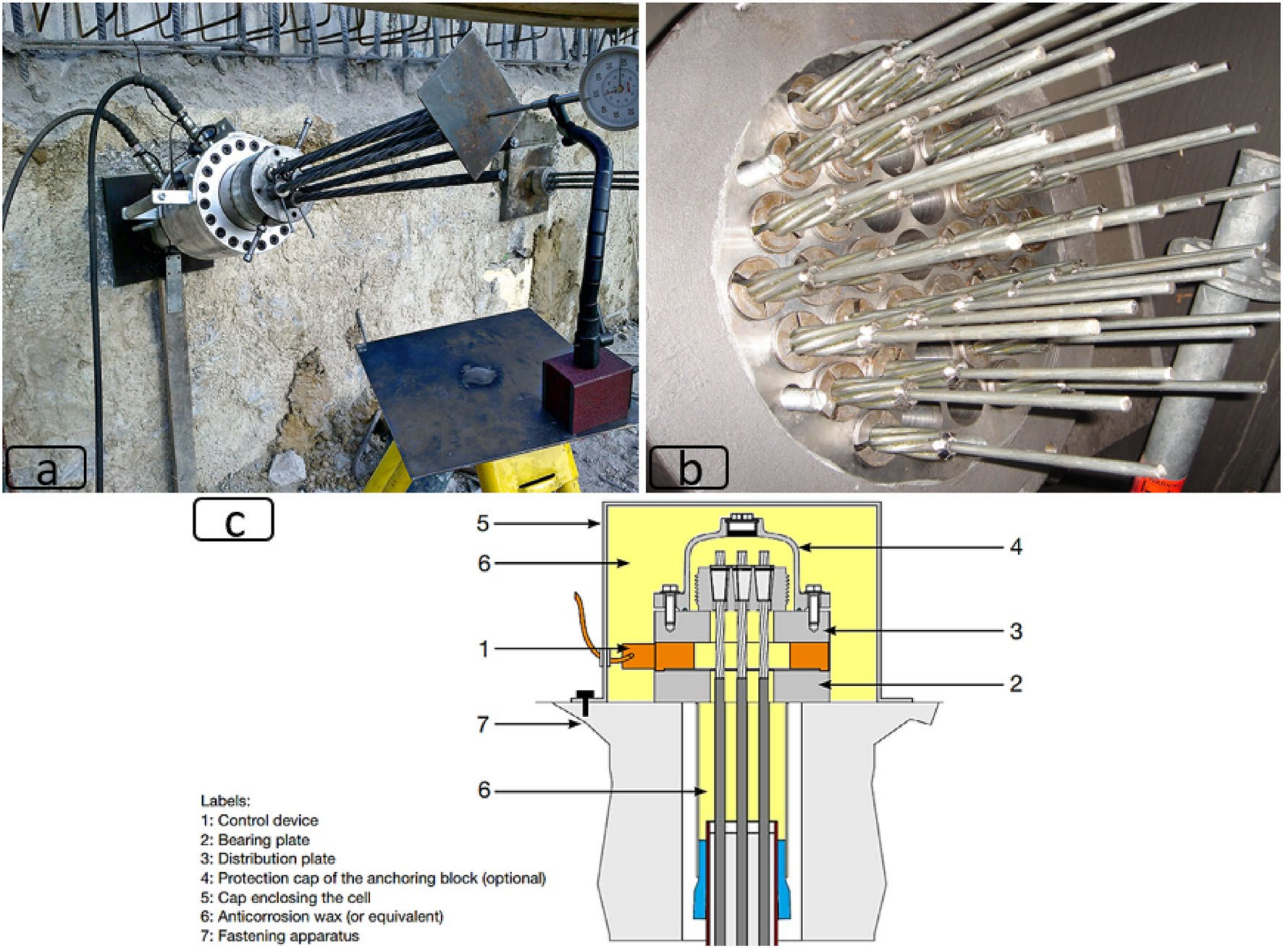
Application of barrel and wedge in other disciplines, (**a**) soil anchors^7^, (**b**) cable and suspension bridges^8^, (**c**) schematic of an instrumented anchor head to monitor axial load over time^9^.

Outside the mining industry, barrel and wedge is widely used in civil and mechanical engineering applications such as grips for machines, cable bridges, suspension bridges, soil nails/anchors, pre/post-tensioned concrete fabrication, coupling cables, etc. In most of these applications, multiple wedges are fixed inside a multi-cable “wedge plate” or “anchor head” (Fig. 2).

Barrel and wedges have some disadvantages. A barrel and wedge becomes a permanent part of the system once activated, and the only practical way of releasing it would be cutting the barrel. Also, compared to rockbolts with a pretensioning nut, pretensioning of cable bolts requires a hollow ram hydraulic jack which utilizes another grip (or barrel and wedge) to react against, making cables longer than usual to accommodate the pretensioning setup (Fig. 1a). This extra length protruding from the borehole, needs to be cut, which due to the complex multi-strand nature of cables, can compromise the cable and even barrel and wedge performance. Like most other termination methods, barrel and wedge are subject to corrosion and material degradation over time.

To the authors' knowledge, no comprehensive dedicated studies on barrel and wedge performance have been conducted, at least in recent years. Most of the knowledge pool returns to the twentieth century. In this research the performance of barrel and wedges are tested in 25 pull out experiments on six different cable bolts in monotonic and cyclic loading patterns. The following sections will detail the experimental plan and methodology proposed to study the barrel and wedge assembly. The results are then presented, and the outcomes are analysed.

## Experiment design

**Figure 3:**
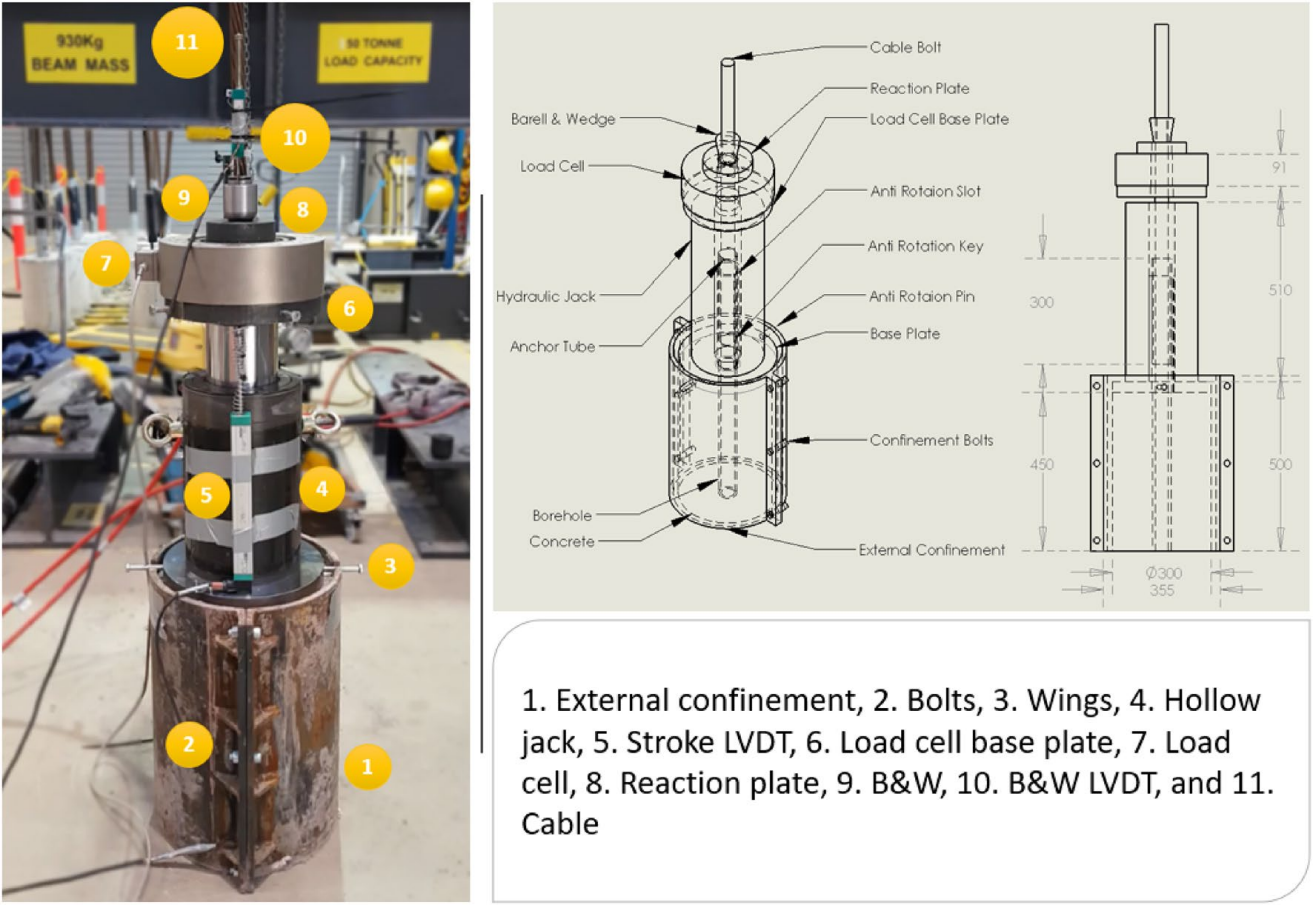
Apparatus schematic and various testing components mid-test^10^.

The barrel and wedges used in this study were utilized in a large-scale laboratory pull out testing campaign^10,11^. The pull out test comprised cables encapsulated in large 300 mm by 450 mm long concrete cylinders with UCS of 40 MPa with approximate ratio of 1.0:1.5:3.0:0.6 (cement:sand:aggregate:water) with a slump of circa 100 mm. In such experiments, the borehole surface is riffled to mimic field condition and promotes failure on the cable/bonding agent interface^12,13^. The concrete cylinders were confined by a thick metal pipe to provide outer confinement and maintain integrity during the tests. On top of the cylinders, a base plate distributed the loads from the 1000 kN hollow ram jack while reacting against the barrel and wedge (Fig. 3).

**Figure 4:**
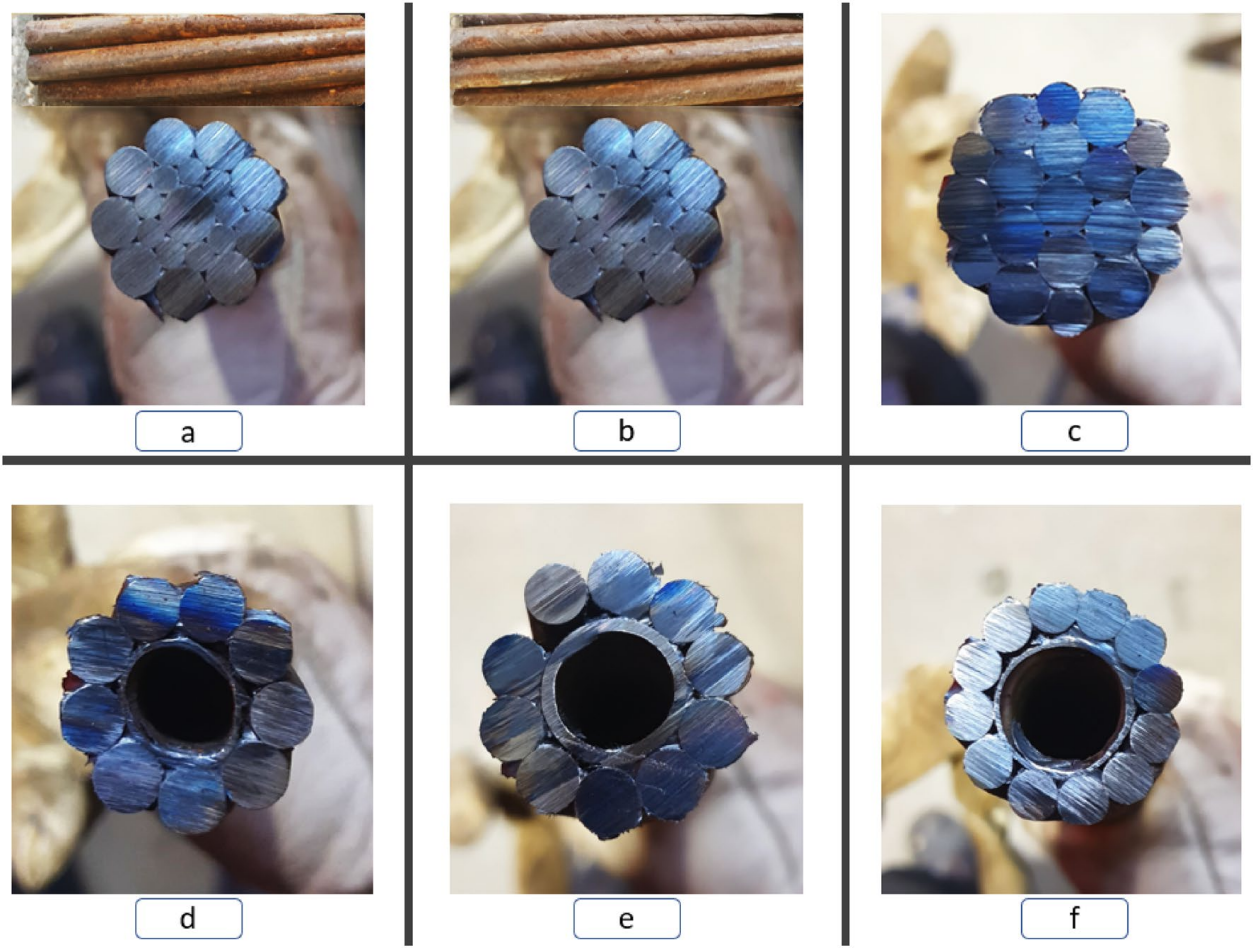
Cable cross-sections—(**a**) Superstrand, (**b**) indented Superstrand, (**c**) Goliath, (**d**) 9 strand SUMO, (**e**) 10 strand SUMO, and (**f**) 12 strand SUMO^10^.

Care was taken in choosing the cables for this study in order to represent a variety of the major cable designs used in practice. *Superstrand* and *Indented Superstrand* cables are relatively smaller and without a bulb. *Goliath* cable is a thicker and heavy unbulbed cable. Furthermore, *9-strand*, *10-strand*, and *12-strand* cables were also used in the experiment. These cables had bulbs along their length (one inside the concrete cylinder and one inside the anchor tube), which governs their behaviour. Tables 1 and 2 showcase the cables and the respected barrel and wedges. Furthermore, Fig. 4 illustrates the cross-section of the cables. Note that 9, 10 and 12 strand cables are hollow core due to the existence of grout tubes.

**Table 1:** Specifications of the cables^1^.

Cable type	Cable diameter (mm)	Breaking point @strands (kN)	Breaking point @B&W (kN)	Steel area (mm^2^)	Elongation at failure (%)	Bulb diameter (mm)
Goliath	28.6	970	> 800	532	5–7	28.6
12 strand SUMO	31	705	640	–	5–7	36
10 strand SUMO	31	705	640	–	5–7	36
9 strand SUMO	28	635	540	–	5–7	35
Indented Superstrand	21.8	590	520	313	6–7	–
Superstrand	21.8	570	450	313	6–7	–

**Table 2:** Barrel and wedge properties^1^.

Name	Cable	Cable diameter (mm)	Barrel length (mm)	Barrel diameter (mm)
31 mm	S10, S12	31	74	70
21.8 mm	SS, IDS	22	60	58
28.6 mm	Gol, S9	28.6, 28.0	73	75

## Testing procedure

**Figure 5:**
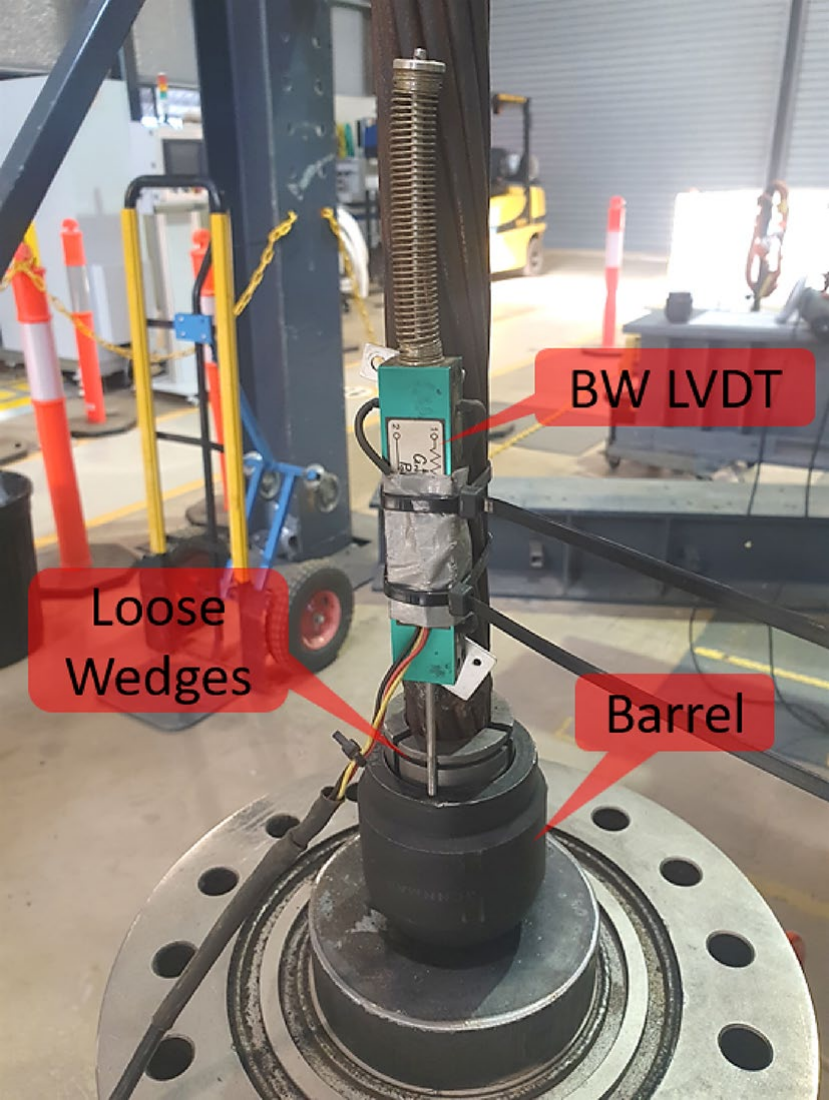
Top of the sample configuration.

Prior to the experiment, the concrete samples were grouted inside the metal outer confinement. After curing, the anti-rotation base plate was lowered onto the top of the sample, a 1000 kN hollow ram jack connected to a low-speed electric hydraulic pump, and a 1000 kN load cell. On top of the load cell, a reaction plate was placed to transfer the load to the barrel and wedge. Before the commencement of the test, the wedge was hammered into the barrel to the extent that more hammering would have resulted in cracking/breakage of the wedge (Fig. 5). This method, though non-scientific, ensure that the slippage of the wedge during the experiment was minimized in the laboratory.

A 225 mm LVDT was fixed to the side of the jack to measure the total vertical displacement in the test, while a 150 mm LVDT was fixed to the cable to measure the displacement of the barrel relative to the cable (Fig. 5). The displacement read by this sensor includes the potential slippage of the wedge plus the barrel displacement during the test. The two LVDTs and the load cell data were recorded using a data acquisition system at 10 Hz.

In total, 25 tests were conducted on the cables, of which 18 were done in a monotonic manner, and seven were carried out in a cyclic manner. The cyclic loading pattern consisted of five equally spaced fully unload/reload cycles during otherwise monotonic loading. To define the unloading load values, the average peak load value from the monotonic test of each cable was divided by five. Thus, the loading steps were 1/5, 2/5, 3/5, 4/5 and 5/5 of the average monotonic load. After the last unload/reload, the sample loaded until failure. The sample may fail before the last unload/reload cycle.

**Figure 6:**
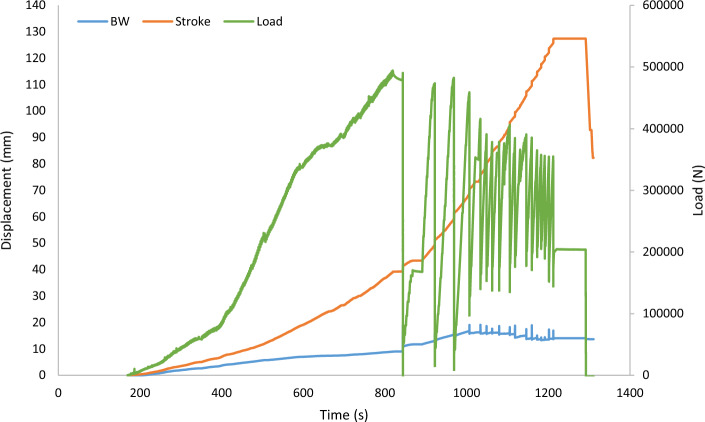
Typical barrel and wedge LVDT (BW), stroke LVDT (Stroke), and load cell (Load) readings over time for 10-strand cable.

Both monotonic and cyclic tests were capped at 120 mm of vertical displacement. Figure 6 illustrates a typical output from the three data channels (i.e., ram stroke LVDT, barrel and wedge LVDT and load cell) during a monotonic loading test. As seen, during the test as the load is increased (in this case monotonically), the cable pulled out length is recorded via the ram stroke LVDT. In Fig. 6, the stroke LVDT values are the sum of pull out length, cable elastic elongation, and barrel and wedge movement. The barrel and wedge LVDT value is the sum of slippage of the wedge on the cable and the penetration of the wedge inside the barrel.

## Result analysis

### Performance under monotonic loading

In this section, the results of the testing campaign are plotted as the *total load of the experiment* (*from the load cell*) versus *the barrel displacement* (*from the BW LVDT*) up until the peak load value before sample failure. Plotting the load in kilo Newton and displacement in millimetres means that the area beneath each graph correlates to the energy (Joule) spent on the barrel and wedge to make reversible and irreversible deformations and displacements under the applied load. In the following figures, the prefix M and C denote *Monotonic* and *Cyclic* loading. SS and IDS stand for *Superstrand* cables (plain and indented) and Gol stands for *goliath* cable. 9S, 10S, and 12S represent the bulbed cables.

**Figure 7:**
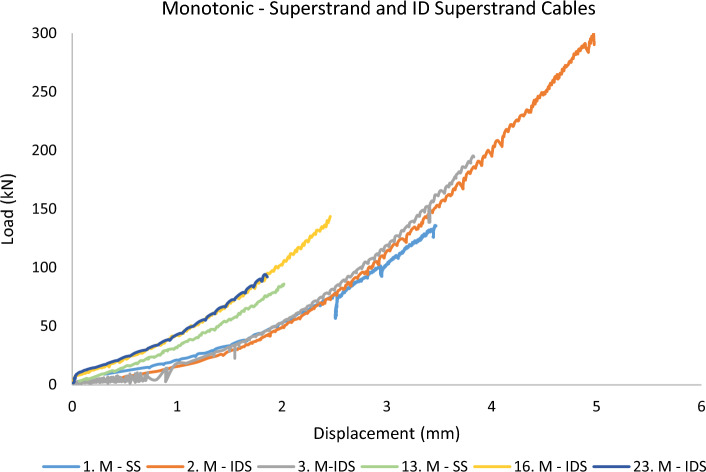
Barrel and wedge performance for Superstrand and Indented Superstrand in monotonic loading.

As seen in Fig. 7, there is no conclusive trend for displacement values measured for *smooth* Superstrand and *Indented* Superstrand cables. The overall behaviour of the system resembles a polynomial of the order of two. Most importantly, though, the graph clearly shows that a displacement of the barrel existed during the whole experiment and never stopped.

**Figure 8:**
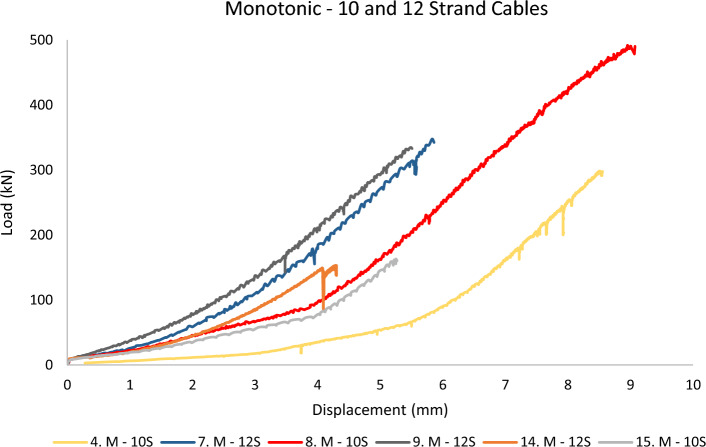
Barrel and wedge performance for 10 and 12-strand cables in monotonic loading.

Figure 8 showcases the 10 and 12-strand cables during the monotonic test. The plots suggest that although the barrel and wedges used for these two cables are identical, the slight difference in the shape of the cable (lay angle and lay length due to strand count) has resulted in variation in the expected behaviour. The 10-strand cable shows a deflection point at a certain displacement at which the behaviour suddenly becomes significantly stiffer. The 12-strand cable, on the other hand, behaves similarly to the Superstrand cables, albeit at higher load and displacement. Interestingly, up to 9 mm of displacement is observed for up to 500 kN of load.

**Figure 9:**
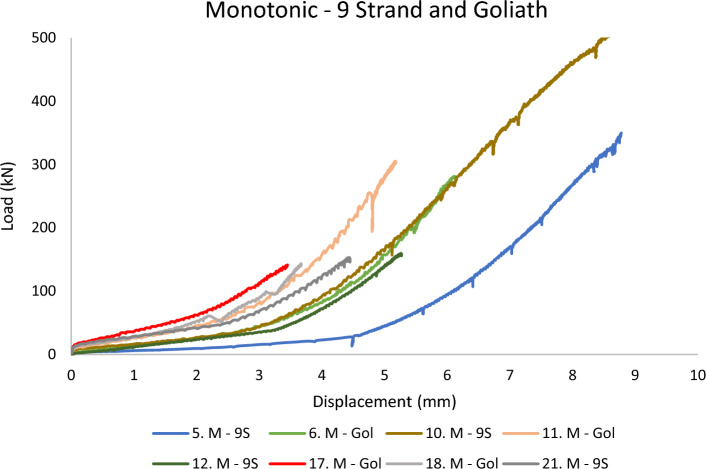
Barrel and wedge performance for 9 strand and Goliath cables in monotonic loading.

The performance of 9-strand and Goliath cables is portrayed in Fig. 9. Similar to the other cables, large displacements are visible throughout the experiments. In saying that, the behaviour seems to be more in tangent with the 10-strand cables than the Superstrand cables. The common point of the 9, 10, 12-strand and Goliath cables is their larger diameter (Tables 1 and 2). This suggests the diameter of the cable can be of importance for the performance of the barrel and wedge (except for 12 strand). The deflection point in the behaviour of these cables proposes that most of the displacement occurs in the lower loads for these cables. As mentioned above, the hollow core 9-strand cable and full core goliath cable have shown the deflection point-based characteristic, suggesting the grout tubes in the middle of the bulbed cables have a minimal adverse effect on the barrel and wedge behaviour.

## Cyclic performance

**Figure 10:**
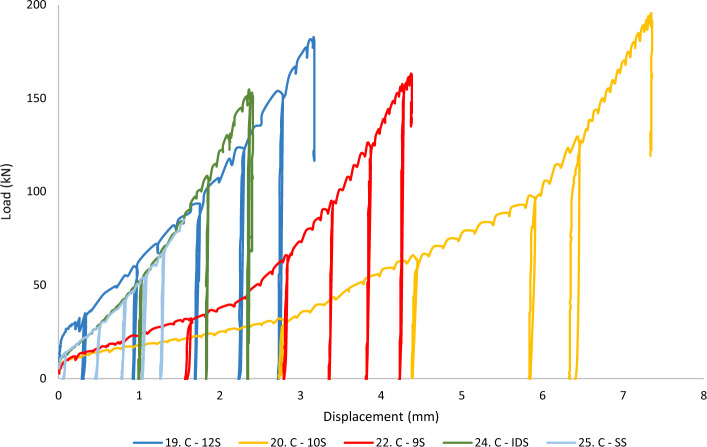
Performance of cable bolts in cyclic loading.

Figure 10 illustrates the barrel and wedge assembly response in the cyclic tests. As seen, in all cables regardless of the amount of displacement and load, the cables had almost instantaneous rebound after the unloading steps. This is seen by the vertical load pickups and suggests that minimum elastic displacement (energy) is stored in the system. Barrell and wedges need to be able to withstand various loading events due to the unpredictable and dynamic environments of underground mines, where changes in stress regime, blasting and seismic events such as quakes or rockbursts are often common.

Another point worth mentioning is the observation of the deflection point based behaviour for the 9 and 10 strand cables, while the Superstrand and 12-strand cables illustrate slight exponential behaviour (similar to the monotonic tests). The 9 and 10-strand cables show significantly larger displacements for a given load compared to the rest of the cables tested, a phenomenon more or less spotted in the monotonic experiments. Interestingly, the 12-strand cable (a bulbed, large, heavy cable) performed very similarly to the Superstrand and Indented Superstrand cables (unbulbed smaller cables).

## Conclusions

This study presented findings on the performance of barrel and wedge assembly used in cable bolting. Dedicated LVDT, fixed on the free end of the cable, measured the relative displacement of the barrel during large-scale laboratory monotonic and cyclic pull out tests. Multiple bulbed and unbulbed cables were tested, and the results suggested the followings:

- Similar cables in diameter can possess different performances as seen by the 10 and 12 strand cables. The difference between these two cables was in the lay angle and lay length, with 10 strand cable having a longer lay length. As a result, the 10 strand cables showed much higher displacement for a given load value compared to the 12 strand cables. Moreover, 10 strand cables had a distinct deflection point at around 4–5 mm displacement, at which the behaviour suddenly became stiffer. We know for a given barrel and wedge and cable, the longer the lay length, the larger the contact area of a single strand with a single wedge. On the contrary, each strand has a higher chance to be in touch multiple wedges for the same cable with a shorter lay length.
- The deflection point-based behaviour was also witnessed in the 9 and 10 strand and Goliath cable. This suggests larger cables tend to behave similarly while smaller cables have exponential behaviour. In saying this, however, 12 strand cable showed exponential behaviour in monotonic and cyclic tests.
- The 9, 10 and 12 strand cables all have a metal grout tube in the centre which were kept hollow for the experiments. Comparing their performance with the full core large Goliath cable advises the grout tube does not affect the results in a meaningful way.
- All cables showed reasonable performance in the cyclic loading with almost vertical load pickup, meaning all the displacement of the barrel and wedge assembly for all the loads larger than 25 kN were permanent. The 9 and 10 strand cables showed higher displacement than the 12 and Superstrand cables, a behaviour also seen more or less in the monotonic tests.

As shown, barrel and wedge can influence the design of the cable bolts. For any given displacement, various load values could be expected. Nevertheless, one should acknowledge that the values in the graphs were concurrent with up to 120 mm of total pull out displacement. This makes the < 10 mm of the barrel and wedge displacement only a fraction (< 10%) of the total displacement of the system. Moreover, as seen in the cyclic tests, the energy seems to be spent on the permanent deformation of the barrel and wedge assembly.

It is suggested that more dedicated and comprehensive studies be conducted on barrel and wedges, perhaps in more loading variations such as instantaneous loading (drop tests) or fatigue testing (seismicity and blasting). Moreover, corrosion studies can also be helpful in indicating how long a barrel and wedge can act *as intended in the design* over time.

Perhaps, a unified way of testing both in the laboratory and field can be helpful. Another limitation observed in this study was the absence of a set way of initializing the assembly to the cable. In this study, this was done by hammering the wedge inside the barrel until no further displacement was allowed. This method was done to make sure no excessive slippage happened at the beginning of the tests. However, this technique was far from scientific and repeatable.

### Supplementary Information

Supplementary Information.

## Data Availability

All data generated or analysed during this study are included in this published article [and its supplementary information files].
